# Admission of advanced lung cancer patients to intensive care unit: A retrospective study of 76 patients

**DOI:** 10.1186/1471-2407-11-159

**Published:** 2011-05-02

**Authors:** Claire Andréjak, Nicolas Terzi, Stéphanie Thielen, Emmanuel Bergot, Gérard Zalcman, Pierre Charbonneau, Vincent Jounieaux

**Affiliations:** 1 Service de Pneumologie et Réanimation. Centre Hospitalier Universitaire. Amiens, France; 2 Service de Réanimation Médicale. Centre Hospitalier Universitaire Côte de Nacre. Caen, France; 3 INSERM, ERI27, Caen, F-14000 France; University of Caen, Caen, France; 4 Service de Pneumologie. Centre Hospitalier Universitaire Côte de Nacre. Caen, France

## Abstract

**Background:**

Criteria for admitting patients with incurable diseases to the medical intensive care unit (MICU) remain unclear and have ethical implications.

**Methods:**

We retrospectively evaluated MICU outcomes and identified risk factors for MICU mortality in consecutive patients with advanced lung cancer admitted to two university-hospital MICUs in France between 1996 and 2006.

**Results:**

Of 76 included patients, 49 had non-small cell lung cancer (stage IIIB n = 20; stage IV n = 29). In 60 patients, MICU admission was directly related to the lung cancer (complication of cancer management, n = 30; cancer progression, n = 14; and lung-cancer-induced diseases, n = 17). Mechanical ventilation was required during the MICU stay in 57 patients. Thirty-six (47.4%) patients died in the MICU. Three factors were independently associated with MICU mortality: use of vasoactive agents (odds ratio [OR] 6.81 95% confidence interval [95%CI] [1.77-26.26], p = 0.005), mechanical ventilation (OR 6.61 95%CI [1.44-30.5], p = 0.015) and thrombocytopenia (OR 5.13; 95%CI [1.17-22.5], p = 0.030). In contrast, mortality was lower in patients admitted for a complication of cancer management (OR 0.206; 95%CI [0.058-0.738], p = 0.015). Of the 27 patients who returned home, four received specific lung cancer treatment after the MICU stay.

**Conclusions:**

Patients with acute complications of treatment for advanced lung cancer may benefit from MCIU admission. Further studies are necessary to assess outcomes such as quality of life after MICU discharge.

## Background

Lung cancer is the second most common malignancy (after prostate cancer in males and breast cancer in females) in the USA, and remains the leading cause of cancer-related death in both men and women worldwide [1]. However, the 5-year survival rate (all stages combined) is only 16%, and ranges from 50% in localized cancer to 3% in metastatic cancer [1]. Despite this poor prognosis, patients with lung cancer are increasingly admitted to medical intensive care units (MICUs) for critical illnesses related either to the underlying malignancy (regardless of the cancer stage) or to co-morbidities [2-4]. Previous studies showed poor outcomes in lung cancer patients admitted to the MICU and most notably those requiring mechanical ventilation [3-5]. Nevertheless, overall survival rates in these patients have improved over the last decade [2,6,7]. Three factors may have contributed to this welcome trend: (i) the ever-increasing number of new treatments for solid tumors, (ii) earlier admission to the MICU with the use of new techniques such as non-invasive ventilation (NIV) and aggressive management of septic shock [8,9]; (iii) improved selection of patients likely to benefit from MICU admission [10-12].

However, the patient populations in most of the previous studies [2,3,7,10,12-15] were relatively heterogeneous in terms of disease stage. To the best of our knowledge, very few studies focused specifically on MICU outcomes of patients with advanced lung cancer that is, patients for whom no potentially curative surgical procedure was available. Given the scarcity of healthcare resources, particularly during the current period of economic crisis, careful attention must be directed to allocating resources in compliance with the principle of distributive justice. MICU admission is costly, and selecting patients who are likely to benefit constitutes good husbandry of public resources. In addition, the patient and family should not be unnecessarily exposed to the burden associated with an ICU stay. To select patients for MICU admission, information on factors associated with MICU mortality is needed.

Here, our primary objective was to assess the outcome of patients with advanced lung cancer who were admitted to the MICU. We also looked for factors associated with mortality. To meet these objectives, we performed a multicenter retrospective study of patients admitted to two university-hospital MICUs.

## Methods

This study was performed in the MICUs of the Amiens and Caen University Hospitals (France), which admit 380 and 640 patients per year on average, respectively. Both MICUs are managed by full-time faculty members. The study was approved by the local independent ethics committee called "CEERNI" which is the "Amiens Ethical committee of non interventional research", which is affiliated with CPP Nord Ouest II.

### Patients

We retrospectively reviewed the medical records of lung cancer patients admitted to the study MICUs between January 1996 and December 2006. Consecutive adults (18 years or older) with a previous diagnosis of lung cancer who were admitted to the MICUs during the study period were potentially eligible. MICU admission decisions were made by the senior intensivists often after discussion with the oncologist. Patients with lung cancer diagnosed and/or staged only after MICU admission were not eligible. Among potentially eligible patients, we identified those advanced lung cancer, defined as lung cancer for which no potentially curative surgical options were available, that is, localized or disseminated stage IIIB or IV non-small cell lung cancer (NSCLC) according to the Mountain classification [16] or small cell lung cancer (SCLC)). We excluded patients those lung cancer had been in remission for more than 5 years and those with MICU stay durations shorter than 24 hours, except if they died in the MICU (e.g., after admission at night followed by treatment limitation decisions on the next day). We also excluded patients admitted to the MICU for postoperative care, as the study MICUs admit very few postoperative patients whose characteristics differ considerably from the overall MICU population. For patients admitted more than once to either MICU, only the first admission was considered.

We collected demographic information (age and gender), lung cancer stage and histological type, and cancer treatments (if any) received in the MICU. Within 24 hours after MICU admission, we recorded smoking history, co-morbidities, WHO performance status, the Simplified Acute Physiology Score (SAPS) II, and the Acute Physiology and Chronic Health Evaluation (APACHE) II score [17,18]. For each patient, we computed the Charlson co-morbidity index (CCI) without taking the presence of lung cancer into account [19]. The CCI includes 19 major disease groups. We defined three CCI categories: low (CCI = 0), mid-range (CCI = 1-2), and high (CCI≥3).

### Reasons for ICU admission

We used the clinical and laboratory data in the charts to determine the main reason for MICU admission (infection with or without bone narrow failure, heart failure, non-infectious pulmonary disorder or neurological failure). Laboratory data obtained within 24 h of admission were recorded; they included the hemoglobin level, white blood cell count, platelet count, blood urea nitrogen level, serum creatinine, serum lactate and serum electrolytes, liver function tests, arterial blood gas values and serum and blood results.

We classified reasons for MICU admission into four groups: *i) *reasons unrelated to the lung cancer (e.g. cardiac dysrhythmia, myocardial infarction, or renal infection), *ii) *complications of cancer management (e.g. chemotherapy-induced bone narrow failure, acute heart failure during chemotherapy, or bleeding during bronchoscopy) *iii) *events related to cancer progression (e.g. superior vena cava syndrome), and *iiii) *events induced by the lung cancer (e.g., pulmonary embolism).

### Organ failures at MICU admission

For each patient, we recorded the type and number of organ failures at MICU admission as follows: *(i) *acute renal failure (definition used by Soares et al [4]) as creatinine > 120 μmol/l and uremia >8 mmol/l or creatinine clearance (Cockcroft) <60 ml/min; *(ii) *acute respiratory failure defined as respiratory rate >25/minute, cyanosis, clinical symptoms of respiratory distress, or PaO_2_/FiO_2 _<300 mmHg; *(iii) *hemodynamic failure defined as a need for vasoactive agents (norepinephrine, dobutamine, or epinephrine); *(iv) *neurological failure defined as a Glasgow Coma Scale score< 10 or subjective criteria such as confusion, decreased responsiveness, or coma in the absence of sedation; and *(vi) *bone narrow failure: defined as a white blood cell count <2000/mm^3 ^and/or platelet cell count <100 000/mm^3^, and/or haemoglobin < 10 g/dL. Sepsis was diagnosed using the criteria developed at the American College of Chest Physicians/Society of Critical Care Medicine consensus conferences [20].

### MICU management

We recorded the following data on management in the MICU: use, start time and duration of invasive mechanical ventilation and/or NIV; vasoactive agents; antibiotics and other anti-infectious drugs; hemodialysis; and the anti-cancer treatments.

### Outcome

We determined mortality at the end of the MICU stay and at the end of the hospital stay. Follow-up time was computed from MICU admission to death, discharge or January 1, 2008, whichever came first.

### Statistical analysis

The statistical analysis was performed using SPSS 12.0 software (SPSS Inc., Chicago, IL, USA). Unless otherwise stated, continuous quantitative variables were described as the mean ± standard deviation and ordinal variables as the number and percentage. To identify variables independently associated with death, we performed a univariate comparative analysis using Fisher's exact test for ordinal variables and the Student *t *test or Kruskal-Wallis nonparametric test for continuous variables. Given the long inclusion period (11 years), we checked for a period effect. We also looked for a centre effect. Variables with p-value equal to/or lower than 0.1 by univariate analysis (except SAPS II and Apache II) were then subjected to backward stepwise logistic regression analysis. Variables were formally tested for collinearity. When variables were collinear, we used clinical judgement to select only one variable for inclusion in the model (e.g., we included mechanical ventilation and therefore did not include PaO_2_/FiO_2_, or acute respiratory failure). Seven variables were finally included in the model. The overall fit of our binary regression model was assessed using the Hosmer and Lemeshow chi-square test of goodness of fit. P-values lower than 0.03 (Bonferroni correction for multiple analyses) were considered statistically significant. A univariate survival analysis was performed using the Kaplan-Meier nonparametric method to calculate crude cumulative survival after MICU admission. The end point was April 1, 2009. Percentages were computed using the total number of patients in the overall study population or relevant group as the denominator unless otherwise specified

## Results

### Population

Between January 1, 1996, and December 31, 2006, there were 210 admissions of patients with lung cancer in the two study MICUs. Of these admissions, 134 were excluded from the study, for the following reasons: diagnosis of lung cancer during the MICU stay (n = 45), lung cancer staging during or after the MICU stay (n = 38); previously diagnosed stage I, II, or IIIA NSCLC (n = 43); or repeat admission during the study period (n = 8 admissions in 6 patients).

The remaining 76 patients (69 males) met the inclusion criteria and constituted the study population. Among them, 17 were admitted during the first 6 study years and 59 during the last 5 study years. Table 1 reports the main patient characteristics. Mean age was 63 ± 10 years and mean body mass index (available for 29 patients) was 26 ± 4 kg/m^2^. The diagnosis was NSCLC in 49 patients (stage IIIB in 20 and stage IV in 29) and SCLC in 29 patients (extensive in 18 and localized in 11); 2 patients had both NSCLC and SCLC. NSCLC histology was squamous cell carcinoma in 30 patients, adenocarcinoma in 14, and undifferentiated carcinoma in 5. Co-morbidities were reported in 47 patients including 25 with a mid-range CCI values and 22 with a high CCI values. Median CCI was 1.5 (range 0-7). Before MICU admission, 50 patients received specific chemotherapy and/or radiotherapy for lung cancer (Table 1). The mean time interval from lung cancer diagnosis to MICU admission was 117 ± 154 days (median: 50 days).

**Table 1:** Baseline characteristics of the overall population.

	All patients n = 76	MICU survivors n = 40	MICU non survivors n = 36	*P *value
**Patient characteristics**				

Age (years), mean ± SD	62.95 ± 9.9	62.94 ± 9.7	62.96 ± 10.3	0.99

Males, n (%)	69 (90.8)	36 (90)	33 (91.7)	0.56

Period: before January 1, 2002	17 (22.4)	10 (25)	7 (19.4)	0.36

**Co-morbidities**				

Smoking, n (%)	71 (95.9)	39 (97.5)	32 (94.1)	0.43
CCI, mean ± SD [range]	1.61 ± 1.16 0[1-7]	1.69 ± 1.54 0[1-4]	1.63 ± 1.71 0[1-7]	0.87
CCI Low, n (%)		13 (32.5)	14 (35)	0.83
Mid-range, n (%)		14 (35)	11 (27.5)	
High, n (%)		13 (32.5)	11 (27.5)	
COPD, n (%)	24 (32)	12 (30)	12 (33)	0.42
Chronic respiratory failure, n (%)	4 (5.3)	3 (7.5)	1 (2.7)	0.37
Diabetes, n (%)	9 (11.8)	5 (12.5)	4 (11.1)	0.57
Non pulmonary neoplasm, n (%)	16 (21)	10 (25)	6 (16,6)	0.28

**Lung cancer**				

NSCLC, n (%)	49 (64.5)	28 (70)	21 (58.3)	0.21
Squamous cell carcinoma, n (%)	30/49 (61,2)	18/29 (62.1)	12/20 (60)	0.56
Adenocarcinoma, n (%)	14/49 (28.6)	7/29 (24.1)	7/20 (35)	0.30
Undifferentiated carcinoma, n (%)	2 (4.1)	2/29 (6.9)	0	0.34
SCLC, n (%)	29 (38.2)	14 (35)	15 (41.7)	0.35

Stage*				

NSCLC IIIB, n (%)	20/49 (40.8)	10/28 (35.7)	10/21 (47.6)	0.29
NSCLC IV, n (%)	29/49 (59.2)	18/28 (64.3)	11/21 (52.4)	0.29
Localized SCLC, n (%)	11/29 (37.9)	6/14 (42.9)	5/15 (33.3)	0.44
Disseminated SCLC, n (%)	18/29 (62.1)	8/14 (57.1)	10/15 (66.7)	0.44
Metastasis, n (%)	45 (59.2)	24 (60)	21 (58.3)	0.53

**Lung cancer treatment**				

Chemotherapy, n (%)	43 (56.5)	19 (47.5)	24 (66.7)	0.073
Radiotherapy, n (%)	22 (28.9)	8 (20)	14 (38.9)	**0.05**
Surgery, n (%)	6 (7.9)	2 (5)	4 (11)	0.28

Percentages are relative to the total number of patients (n = 76 patients for the overall population, n = 36 for the MICU non survivors and n = 40 for the MICU survivors) unless otherwise specified. *Two patients had both NSCLC and SCLC.CCI: Charlson Comorbidity Index; NSCLC: Non small cell lung cancer; SCLC: small cell lung cancer; MICU: medical intensive care unit. Metastasis means extensive SCLC or stage IV NSCLC

### Main reasons for MICU admission

The main reasons for MICU admission are listed in Table 2. Acute respiratory failure was the most common organ dysfunction (80%). Admission was also variously due to an infection (41 patients), acute respiratory failure unrelated to infection (28 patients), heart failure (in 7 patients), neurological failure (in 4 patients), and pulmonary embolism (2 patients).

**Table 2:** Reasons for admission to the medical intensive care units in the overall population, nonsurvivors, and survivors

	All patients n = 76	MICU survivors n = 40	MICU nonsurvivors n = 36	*P *value
**Causes of reasons for admissions**				

Not related to lung cancer	16 (21.1)	8 (20)	8 (22.2)	0.51

Related to lung cancer	60 (78.9)	32 (80)	28 (77.8)	0.51
Complication of cancer management, n (%)	30 (39.5)	20 (50)	10 (27.8)	**0.04**
Cancer progression, n (%)	14 (18.4)	7 (17.5)	7 (19.4)	0.53
Lung cancer-induced events, n (%)	17 (22.4)	6 (15)	11 (30.6)	0.089

**Reasons for admission**				

Infection, n (%)	41 (53.9)	15 (37.5)	26 (72.2)	**0.002**
Respiratory infection, n (%)	31 (40.8)	10 (25)	21 (58.3)	**0.003**
Sepsis with aplasia, n (%)	12 (15.8)	6 (15)	6 (16.7)	0.54
Heart failure, n (%)	7 (9.2)	3 (7.5)	4 (11.1)	0.44
Noninfectious pulmonary disorders, n (%)	28 (36.8)	18 (45)	10 (27.8)	0.09
Neurological failure, n (%)	4 (5.3)	3 (7.5)	1 (2.8)	0.35
Pulmonary embolism, n (%)	2 (2.6)	1 (2.5)	1 (2.8)	0.72

MICU: Medical Intensive Care Unit

Percentages are relative to the total number of patients (n = 76 for the overall population, n = 36 for the MICU nonsurvivors, and n = 40 for the MICU survivors) unless otherwise specified.

In 60 patients, MICU admission was directly related to lung cancer. Reasons for MICU admission in these 60 patients were as follows: *(i) *complication of cancer management in 30 patients (adverse events related to lung cancer treatment such as acute heart failure after chemotherapy, chemotherapy-induced bone marrow failure, pneumothorax after central venous catheter insertion, radiation-induced pneumonitis [n = 25] and adverse events related to investigations used to monitor the treatment, such as bleeding during bronchoscopy [n = 5]); *(ii) *cancer progression in 14 patients; and *(iii) *lung cancer-induced events (e.g. pulmonary embolism or hemoptysis) in 17 patients.

Table 3 reports the main clinical and laboratory features in the patients at MICU admission.

**Table 3:** Clinical and laboratory characteristics at admission to the medical intensive care units in the overall population, nonsurvivors and survivors

	All patients n = 76	MICU survivors n = 40	MICU nonsurvivors n = 36	*P *value
SAPS II	43 ± 16.5	40 ± 13	46 ± 19	0.14
Apache II	22 ± 7.7	21 ± 6.8	23.5 ± 8.5	0.11

Blood cell count (/mm^3^)	11616 ± 8688	12899 ± 9250	10226 ± 7931	0.18
Hemoglobin (g/dL)	10.7 ± 2.2	10.5 ± 2.3	10.9 ± 2.2	0.4
**Platelet count (x1000/mm^3^)**	240 ± 236	304 ± 294	173 ± 126	**0.016**
**Platelet count ≤ 100 000/mm^3^**, n (%)	19 (25.7)	4 (10.5)	15 (41.7)	**0.002**
Calcium (mmol/L),	2.1 ± 0.1	2.11 ± 0.19	2.08 ± 0.24	0.54
Albumin (g/L)	25 ± 6.4	27 ± 6	23 ± 6	0.14
**Albumin ≤ 25 g/L, n (%)**	16 (51.6)	4 (30.8)	12 (66.7)	**0.05**
**PaO_2_/FiO_2_**	177 ± 102	201 ± 104	149 ± 93	**0.05**

**Number of organ failures**	2 ± 1.2	1.7 ± 1	2.3 ± 1.3	**0.036**
**Acute respiratory failure, n (%)**	66 (86.8)	30 (75)	36 (100)	**0.001**
**Mechanical ventilation, n (%)**	56 (73.7)	23 (57.5)	33 (91.7)	**0.001**
NIV, n (%)	17 (22.4)	6 (15)	11 (30.6)	0.089
**Invasive ventilation, n (%)**	50 (65.8)	19 (47.5)	31 (86.1)	**<0.0001**
Time on mechanical ventilation (days)	9,8 ± 14	12 ± 17	8,5 ± 12,4	0.41
**Need for vasoactive agents, n (%)**	25 (32.9)	5 (12.5)	20 (55.6)	**<0.0001**
Acute renal failure, n (%)	26 (34.6)	11 (27.5)	15 (41.7)	0.14
hemodialysis, n (%)	7 (9.2)	2 (5)	5 (13.9)	0.17
Neurological failure, n (%)	16 (21.1)	8 (20)	8 (22.2)	0.52

Chemotherapy in the ICU, n (%)	4 (5.5)	1 (2.7)	3 (8.3)	0.29
Corticosteroids in the ICU, n (%)	7 (9.3)	5 (12.5)	2 (5.7)	0.27

MICU: Medical Intensive Care Unit; SAPS II Simplified Acute Physiology Score; Apache II Acute Physiology and Chronic Health Evaluation; NIV: Non invasive ventilation.

Percentages are relative to the total number of patients (n = 76 for the overall population, n = 36 for the MICU nonsurvivors, and n = 40 for the MICU survivors) unless otherwise specified. Quantitative data are expressed as mean ± standard deviation

### Patient management in the MICU

Median [range] MICU stay length was 4.0 [1-178] days (Table 3). During the MICU stay, 57 patients received mechanical ventilation. Of these, 18 (4/17 in 1996-2001 and 14/59 in 2002-2006) received NIV and 50 received invasive ventilation (after failed NIV in 11 patients). The mean duration of invasive ventilation was 9.9 ± 14 days [1-65]. Vasoactive agents were required in 25 patients. Seven patients underwent hemodialysis and 57 received antibiotics. Specific lung cancer treatment was given in 34 cases and consisted of chemotherapy (n = 4), corticosteroid therapy for superior vena cava syndrome (n = 7), growth factor therapy for chemotherapy-induced neutropenia (n = 7), red blood cell or platelet transfusions for chemotherapy-related anemia or thrombocytoaenia (n = 19) and bronchial artery embolization (n = 2).

### Outcomes

**Figure 1:**
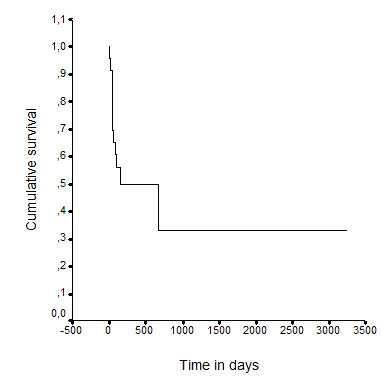
**Cumulative survival of the 27 patients who went back home after the stay in the medical intensive unit**. MCIU = medical intensive care unit. Follow-up is counted from MICU discharge.

Of the 76 patients, 36 died in the MICU (47.4%) and 13 died after MICU discharge but before hospital discharge (49/76, 64.5%). In the 27 patients who returned home, the median [95%CI] survival time after MICU discharge was 157 [0-701] days, with 15 patients alive on day 60 and 10 on day 120 (Figure 1). Two patients were long-term survivors (still alive at last follow-up after 54 and 107 months, respectively). Four patients underwent lung cancer treatment after MICU discharge.

### Factors predicting for MICU mortality

We found no period effect (mortality 41.2% in1996-2001 vs. 49.2% in 2002-2006, p = 0.38) or centre effect (p = 0.15). In the univariate analysis (Tables 1, 2 and 3), age, gender and co-morbidities did not influence MICU mortality. A history of radiotherapy was associated with a significantly higher mortality rate (p = 0.05). Patients with SCLC and those with advanced NSCLC did not differ in terms of the mortality rate. Patients admitted to the MICU for complications of cancer management had a lower mortality rate than patients admitted for other reasons (33.3% vs. 56.5% respectively, p = 0.04). Infection was associated with higher mortality (72.2% vs 37.5% in non-infected patients, p = 0.002). The SAPS II and the APACHE II severity scores did not significantly predict MICU mortality. Laboratory findings that significantly predicted mortality by univariate analysis were albumin level, thrombocytopenia, and PaO_2_/FiO_2_. A need for mechanical ventilation or vasoactive agents was associated with higher mortality rates (59% vs 15%, p < 0.0001) and 80% vs 31% p < 0.0001), respectively). Of the four patients who received chemotherapy in the MICU, three died during their stay.

The multivariate analysis identified three factors independently associated with higher MICU mortality: the use of vasoactive agents (odds ratio [OR] 6.81, 95%confidence interval [95%CI] 1.77-26.26, p = 0.005), requirement for mechanical ventilation (OR 6.61 95% CI [1.44-30.5], p = 0.015) and platelet count below 100,000/mm^3 ^(OR 5.13, 95%CI [1.17-22.5]; p = 0.030). In contrast, admission for a complication of cancer management was associated with lower mortality (OR 0.206, 95% CI [0.058-0.738], p = 0.015).

## Discussion

A large proportion of cancer patients experience acute life-threatening episodes related to their malignancy, treatment or co-morbidities. Until recently, MICU admission of patients with advanced cancer was controversial and generally discouraged [21]. However, over the last decade, several studies found increased survival rates in this population [2,7,8,14,22]. Advances in both oncology and intensive care may have contributed improved survival rates, along with better selection of the patients most likely to benefit from MICU admission [23].

Our study has several limitations. We used a retrospective design and were therefore unable to determine the criteria used to select patients for MICU admission. Consequently, we cannot assess the selection bias induced by MICU triage process. In all likelihood, some patients with advanced lung cancer and life-threatening events were deemed too sick to benefit from MICU admission. Indeed, many of our patients had a WHO performance status of 2 or less. Second, the two study MICUs may have differed regarding their patient selection criteria. Third, patients were included over an 11-year period, during which the management of lung cancer improved. However, we found no evidence of a significant period effect. Finally, our small sample size (n = 76) produced limited statistical power. Indeed, to have a homogeneous population, we should have excluded 134 patients. Unfortunately, we have no data in term of outcome after MICU stay about these 134 patients.

To the best of our knowledge, data on patients with advanced lung cancer admitted to the MICU are scant. Most of the previous studies were done in highly heterogeneous populations [4,13,14]. Our study is the first to focus exclusively on MICU admission of patients with advanced lung cancer and to classify reasons for MICU admission according to their link with lung cancer.

In our highly selected population, we found an MICU mortality rate of 47.4% and an in-hospital mortality rate of 64.5%. These results are in accordance with the data recently reported by Reichner *et al*. (43% MICU mortality and 60% hospital mortality) [24], Soares *et al*. (44% MICU mortality and 60% hospital mortality) [4], and Rocques *et al *[7]. However, our patients had more advanced disease than those in the earlier studies, suggesting that, MICU and hospital mortality rates may have improved as it is noticed the other recent studies (See additional file 1). The number of patients with advanced lung cancer admitted to the study MICUs increased over the study period, from 17 during the first 6 years to 59 during the last 5 years. The absence of a significant period effect may be related to the small sample size.

Mortality rates in lung cancer patients admitted to the MICU have decreased over time. In early studies, mortality ranged from 75% to 91% [5,13,25]. Although Adam *et al*. [2] recently reported an MICU mortality rate of only 22%, only 49% of their patients required mechanical ventilation (versus 75% in our study), and mortality was highest in the mechanically ventilated patients. Moreover, the study by Adam *et al *included patients with all stages of lung cancer.

In our study, MICU mortality was not significantly influenced by age, gender or co-morbidities). A history of thoracic radiotherapy was associated with a significantly higher mortality, suggesting a link between radiation-induced pneumonitis and acute respiratory failure. In contrast to a report by Reichner *et al *[24] that stage IV NSCLC was independently associated with higher mortality, we found no significant influence on MICU mortality of the lung cancer characteristics (e.g., NSCLC vs. SCLC, NSCLC histology, and type of metastasis). Neither did we find any significant difference in mortality between patients who had recently started cancer treatment and patients treated for longer periods.

In line with the previous studies, we found that short-term mortality was mainly related to the severity of organ dysfunction and not to the characteristics of the malignancy [2,4,5,12,13,24]. In our univariate analysis, the main predictors of mortality were the number of organ failures, need for vasoactive agents, and need for invasive mechanical ventilation. Admission for an infection was also associated with higher mortality. This finding may reflect patient selection, as patients with treatment-limitation decisions were not admitted to the MICUs as such EOL care measures are implemented only on the wards in our institutions, and as all patients who were admitted at night and who had treatment-limitation decisions taken the next day were excluded.

As reported elsewhere, the SAPS II and APACHE II score did not significantly predict MICU mortality in our patients with advanced lung cancer: similar results have been reported for the SAPS III and APACHE III score [2,7,26]. These scores underestimate mortality and do not reliably predict MICU mortality in patients with malignancies. Interestingly, several laboratory characteristics were associated with higher mortality in our study. In the multivariate analysis, thrombocytopenia was significantly associated with higher mortality.. We have no explanation to this finding. The medical charts did not contain enough information to determine the causes of thrombocytopenia in our patients. Moreau *et al*. also reported that thrombocytopenia had prognostic significance in a large cohort of MICU patients [27], and the platelet count is included in the SOFA score. Moreover, thrombocytopenia is considered a marker of cancer-related coagulation abnormalities [28,29]. We believe that the platelet count should be included in new scoring systems, as classic severity scores are notoriously unreliable.

The other two variables available at admission and independently associated with MICU mortality by multivariate logistic regression were a need for vasoactive agents and a need for mechanical ventilation. Admission for complications of cancer management was independently associated with lower MICU mortality. One possible explanation is that these complications are acute events that often respond promptly to treatment. An example is acute heart failure during chemotherapy.

In addition to data on mortality, we need information on outcomes in survivors after MICU and hospital discharge. Postdischarge survival is strongly dependent on the malignancy and its treatment. At MICU discharge, a recovery period of several weeks is often needed before chemotherapy can be given. In our study, median survival in the 27 patients who returned home was 157 days, with only 15 patients alive on day 60 and 10 on day 120. However, 2 patients achieved long-term survivals. We did not assess postdischarge quality of life, which was probably severely impaired. Of 10 patients who were potentially eligible for further cancer chemotherapy, 6 had WHO performance status scores greater than 2; thus, only 4 patients received further chemotherapy. These data emphasize the need for appropriate patient selection to MICU admission and, consequently, they have ethical implications. Ideally, outcome evaluations in future studies should include parameters other than mortality, such as quality of life and eligibility for cancer treatment after the MICU stay.

## Conclusions

In conclusion, we found that, the only factor predicting lower mortality was admission for complications of lung cancer treatment. MICU mortality was not influenced by the type of cancer (SCLC or NSCLC) but increased with the severity of the organ failures. Of 27 hospital survivors, only 4 received further specific treatment for lung cancer. Our findings suggest that patients with advanced lung cancer and non-infectious iatrogenic complications may benefit from MCIU admission. Further studies of patients selected to MICU admission are needed to assess long-term mortality, quality of life, ability to continue chemotherapy and socio-economic cost.

## Abbreviations

APACHE II: Acute Physiology and Chronic Health Evaluation; CCI: Charlson Co-morbidity Index; CI 95%: Confidence Interval 95%; MICU: Medical Intensive Care Unit; NIV: Non-Invasive Ventilation; NSCLC: Non-Small Cell Lung Cancer; OR: Odds Ratio; SAPS II: Simplified Acute Physiology Score; SCLC: Small Cell Lung Cancer; WHO performance status: World Health Organization performance status.

## Competing interests

The authors declare that they have no competing interests.

## Authors' contributions

CA contributed substantially to the conception and design of the study, to the collection, and to the analysis. She contributed too to the interpretation of the data; and drafted the manuscript. CA gave final approval of the version to be submitted for publication. NT contributed substantially to the collection, analysis, and interpretation of the data; and drafted the manuscript. NT gave final approval of the version to be submitted for publication. ST contributed substantially to the collection, analysis, and interpretation of the data; and drafted the manuscript. ST gave final approval of the version to be submitted for publication. EB contributed substantially to the conception and design, carefully revised the manuscript and gave final approval of the version to be submitted for publication. GZ contributed substantially to the conception and design, carefully revised the manuscript and gave final approval of the version to be submitted for publication. PC contributed substantially to the conception and design, carefully revised the manuscript and gave final approval of the version to be submitted for publication. VJ contributed substantially to the conception and design, carefully revised the manuscript and gave final approval of the version to be submitted for publication.

## Authors Information

The work has been presented in part at the annual congress of the European Respiratory Society (ERS) held in October 2008, Berlin, Germany.

CA, NT, PC and VJ work in ICUs; ST, EB and GZ provide care of lung cancer patients.

## Pre-publication history

The pre-publication history for this paper can be accessed here:

http://www.biomedcentral.com/1471-2407/11/159/prepub

## Supplementary Material

Additional file 1 **Table S1 shows mortality rates in previous studies of patients with advanced lung cancer admitted to the medical intensive care unit**. * Predictors of ICU outcome; ** Predictors of hospital outcome; *** This study included several times the same patients, and focused on weaning of mechanical ventilation; # univariate analysis; ## multivariate analysis; PS = Performans status; SOFA = Sequential Organ Failure Assessment; APACHE: Acute Physiology And Chronic Health Evaluation; NR: not reported; MV.: Mechanical ventilation. Click here for file
