# Supplementary material for: Admission of advanced lung cancer patients to intensive care unit: A retrospective study of 76 patients
Source: BMC Cancer. 2011 May 2;11:159. doi: 10.1186/1471-2407-11-159 (PMC3112156; doi:10.1186/1471-2407-11-159)
Supplement: Additional file 1 — Table S1 shows mortality rates in previous studies of patients with advanced lung cancer admitted to the medical intensive care unit. * Predictors of ICU outcome; ** Predictors of hospital outcome; *** This study included several times the same patients, and focused on weaning of mechanical ventilation; # univariate analysis; ## multivariate analysis; PS = Performans status; SOFA = Sequential Organ Failure Assessment; APACHE: Acute Physiology And Chronic Health Evaluation; NR: not reported; MV.: Mechanical ventilation. [file 1471-2407-11-159-S1.DOC]

Table S1: Mortality of patients with advanced lung cancer admitted in MICU

|  | Study year | Patients n | III B /IV NSLC, n | SCLC,n | Advanced lung cancer, (%) | MV (%) | ICU mortality (%) | Hospital mortality, (%) | Predictors of outcome |
| --- | --- | --- | --- | --- | --- | --- | --- | --- | --- |
| Ewer et al (25) | 1986 | 46 |  |  |  | 100 | 85 | 91 | MV duration |
| Boussat et al (13) | 2000 | 57 | 43 | 0 | 75 | 91 | 67 | 75 | * # Acute pulmonary disease, Karnofsky PS<70 |
| Jennens et al (10) | 2002 | 20 | 0 | 20 | 100 | 45 | NR | NR |  |
| Lin et al (5) | 2003 | 81 ** | 73 | 22 |  | 100 | 73 | 85 | ** ## Albumin, Apache III, FiO_2_, PEEP, organ failure, ability to shift to partial ventilatory support, MV duration |
| Reichner et al (24) | 2006 | 47 | 33 | 7 | 85 | 49 | 43 | 60 | * ## MV, advanced lung cancer stage, SOFA score |
| Soares et al (26) | 2007 | 143 | 84 | 25 | 76 | 70 | 44 | 60 | ** # Airway infiltration or obstruction by cancer, number of organ failures, cancer recurrence or progression, severity of comorbidities |
| Adam et al (2) | 2008 | 139 | 84 | 18 | 73 | 49 | 22 | 40 | *# Use of vasopressors, 2 or more organ failures |
| Rocques et al [7] | 2009 | 105 | 68 | 18 | 82 | 41 | 43 | 54 | PS>2 and acute respiratory failure |
| Our study |  | 76 | 49 | 29 | 100 | 75 | 47 | 64 | * # Vasopressive drugs, MV, thrombocytopaenia, one good prognosis factor: admission for an adverse event related to lung cancer treatment |

* Predictors of ICU outcome; ** Predictors of hospital outcome; *** This study included several times the same patients, and focused on weaning of mechanical ventilation; # univariate analysis; ## multivariate analysis; PS = Performans status; SOFA=Sequential Organ Failure Assessment; APACHE: Acute Physiology And Chronic Health Evaluation; NR: not reported; MV.: Mechanical ventilation
